# Genetic variation in leaf bronzing and dry matter production of rice varieties as indicators of tolerance to iron toxicity

**DOI:** 10.1270/jsbbs.24081

**Published:** 2025-07-31

**Authors:** Asami Tomita, Juan-Pariaska Tanaka, Matthias Wissuwa, Yoshimichi Fukuta

**Affiliations:** 1 Graduate School of Environmental and Life Science, Okayama University, 1-1-1 Tsushima-Naka, Kita-ku, Okayama 700-8530, Japan; 2 Japan International Research Center for Agricultural Sciences (JIRCAS), 1-1 Ohwashi, Tsukuba, Ibaraki 305-8686, Japan

**Keywords:** iron toxicity, tolerance, genetic variation, association analysis, rice

## Abstract

Iron toxicity, caused by excessive iron uptake, can reduce biomass and yield in rice (*Oryza sativa* L.). In this report, we present a wide genetic variation among 91 varieties, including 18 upland NERICAs, in terms of leaf bronzing score (LBS), dry weight under iron toxicity (Iron-DW), dry weight under control conditions (Control-DW), and relative dry weight (RDW = Iron-DW/Control-DW), using agar nutrient solution at rice seedling stage. We found no correlations between LBS and the other three. These were classified into three clusters: A, B1 and B2, based on these trait variations. Cluster A consists mainly of lowland Japonica and Indica Groups, exhibiting the lowest LBS and RDW that were intermediate between B1 and B2. Clusters B1 and B2 included both Japonica and Indica Groups’ varieties, as well as the 18 upland NERICAs. B1 displayed the highest RDW and a LBS that was intermediate between A and B2. Conversely, B2 had the highest LBS and the lowest RDW. Both the Indica and Japonica Groups displayed extensive variation in tolerance to iron toxicity. Moreover, LBS and RDW are controlled by different genetic mechanisms. In our association analysis using NERICAs, we identified two quantitative trait loci (QTLs): one for LBS on chromosome 9, which is a novel discovery, and another for Control-DW, which enhances tolerance through *O. glaberrima* alleles.

## Introduction

Iron toxicity in rice (*Oryza sativa* L.) is a serious problem, due to excessive iron uptake in lowland cultivation (Dobermann and Fairhurst 2000, Ponnamperuma *et al.* 1955), and reduces biomass and yield (Aboa and Dogbe 2006, Audebert and Fofana 2009). In lowland cultivation, conditions such as flooding, high temperatures, and decomposition of organic matter promote soil reduction, converting ferric iron into ferrous iron, which causes iron toxicity (Becker and Asch 2005). Damage is reported mainly in the tropics, because soil reduction is facilitated in lowland fields by climatic conditions and the short fallow period, and soil improvement is costly (Nozoe *et al.* 2003). Breeding for tolerance to iron toxicity is one way to ensure stable rice production in lowland cultivation in the tropics.

Traits used to evaluate tolerance to iron toxicity in rice include leaf bronzing score (LBS) (Asch *et al.* 2005, Dufey *et al.* 2009, 2012, Elec *et al.* 2013, Engel *et al.* 2012, Fukuda *et al.* 2012, Matthus *et al.* 2015, Shimizu *et al.* 2005a, Wan *et al.* 2003a, 2003b, Wang *et al.* 2008, Wu *et al.* 1998, 2014, Yamauchi *et al.* 1982), dry weight (DW) (Dufey *et al.* 2009, 2012, Fukuda *et al.* 2012, Shimizu *et al.* 2005a, Wan *et al.* 2003a, 2003b, Wang *et al.* 2008, Wu *et al.* 2014), tiller number (Matthus *et al.* 2015, Wan *et al.* 2003b), shoot or root Fe concentration (Asch *et al.* 2005, Dufey *et al.* 2009, 2012, Fukuda *et al.* 2012, Matthus *et al.* 2015, Shimizu *et al.* 2005a, Wang *et al.* 2008, Wu *et al.* 1998, Yamauchi *et al.* 1982), P or K concentration (Shimizu *et al.* 2005a), and chlorophyll content (Dufey *et al.* 2009, 2012). Among them, LBS is a typical visual symptom (Asch *et al.* 2005, Becker and Asch 2005). Significant correlations of LBS were found with grain yield under iron toxicity (Audebert and Fofana 2009, Diop *et al.* 2020, Elec *et al.* 2013, Melandri *et al.* 2021, Sikirou *et al.* 2015, Yamauchi 1989) and with DW under hydroponic conditions (Dufey *et al.* 2009, 2012, 2015a, 2015b, Wan *et al.* 2003b, Wu *et al.* 1998).

Takehisa *et al.* (2004) found QTLs for shoot traits on chromosomes (chrs.) 1, 2, 3 and 7 in a highly reduced lowland field flooded with salt water. Then, Takehisa *et al.* (2006) found QTLs for LBS in the same field using the same hybrid population on a different region on chr. 3. These results indicated that QTLs for LBS and shoot traits were found in different chromosome regions, indicating that these tolerances were controlled by different genetic mechanisms. However, the relationships between genetic mechanisms for LBS and dry weight in shoots have not been clarified yet. To clarify the genetic mechanism for tolerance to iron toxicity for the development of tolerant materials, it may be important to consider the relationships between LBS and dry weight as separate indicators of tolerance. LBS is due to the accumulation of oxidized polyphenols in the leaf blades, following a decrease in the tolerance of symplastic tissue (Becker and Asch 2005). Mechanisms of tolerance to iron toxicity can be divided into oxidation in the roots and Fe selectivity, retention of Fe in roots and stems, and symplastic tissue tolerance. LBS and dry weight under iron toxicity might not necessarily be due to the same mechanisms. Thus, the relationship between LBS and dry weight should be clarified physiologically and genetically.

To clarify the genetic basis of tolerance to iron toxicity, researchers have screened a large number of rice varieties in the iron-toxic swamp or acid sulfate soils in the field (Audebert and Fofana 2009, Mahadevappa *et al.* 1979, Ponnamperuma and Solivas 1981, Rosmini and Mukhlis 1990, Sahrawat and Sika 2002, Virmati 1977) and in hydroponic solution (Asch *et al.* 2005, Dufey *et al.* 2009, 2015a, 2015b, Elec *et al.* 2013, Engel *et al.* 2012, Fukuda *et al.* 2012, Matthus *et al.* 2015, Ouyang *et al.* 2007, Shimizu *et al.* 2005a, 2005b, Wan *et al.* 2003a, 2003b, Wu *et al.* 1998, 2014, Yamauchi *et al.* 1982), and then investigated varietal differences in tolerance. However, the results in hydroponic conditions did not necessarily reproduce those in field conditions (Nozoe *et al.* 2008). For example, The *Oryza glaberrima* cultivar CG 14 was assessed as moderately tolerant in solution (Asch *et al.* 2005) but tolerant in the field (Audebert and Fofana 2009, Sahrawat and Sika 2002). Moreover, the results of several varieties in hydroponic solution varied widely among reports (Fukuta and Wissuwa 2011, Mahender *et al.* 2019). For example, Wan *et al.* (2003b) evaluated IR 26 as sensitive in solution but Yamauchi *et al.* (1982) reported it as tolerant.

One of the main reasons for these contradictory results is thought to be due to the chemical condition of the solution because ferrous iron is easily oxidized in solution (Wang *et al.* 2008). To keep conditions stable in the solution, some researchers modified the solution by lowering its temperature (Shimizu *et al.* 2005b) and adjusting pH and the molar ratio of FeEDTA in the solution (Elec *et al.* 2013) or adding N_2_ gas into it (Wu *et al.* 2014). Even so, the reason for the contradictory results is unclear. Another suggested reason is that the degree of stress on plants might affect tolerance. For example, Suakoko 8, evaluated as sensitive in hydroponic solution (Yamauchi *et al.* 1982) but tolerant in the field (Audebert and Fofana 2009), showed LBS immediately after iron toxicity treatment, but it survived for a long time (Elec *et al.* 2013). Elec *et al.* (2013) also reported that the LBS score in the field was better correlated with grain yield in long hydroponic treatment than in short treatment. Thus, differences in the response to the degree of stress, including treatment duration and iron concentrations, might explain contradictory results between field and hydroponic conditions. Therefore, to assess the genetic variation in tolerance to iron toxicity corresponding with that in the field, we need to investigate the variation in tolerance to different iron concentrations and treatment durations in hydroponic solutions under controlled chemical conditions.

Agar nutrient solution proved to be a suitable tool for screening tolerance to iron toxicity because of its similar pH, Eh, and viscosity to those of the rhizosphere (Wang *et al.* 2008). Yet the degree of tolerance in the solution corresponded with that in the field in only five cultivars. However, Wang *et al.* (2008) did not investigate which stress conditions are suitable for assessing wide variations in tolerance and differences in response under different iron concentrations and durations.

Many QTLs for tolerance to iron toxicity have been detected on all chromosomes using hydroponic solutions (Dufey *et al.* 2009, 2012, 2015a, 2015b, Shimizu *et al.* 2005a, Shimizu 2009, Wan *et al.* 2003a, 2003b, Wu *et al.* 1997, 1998, 2014) or by genome-wide association study (Matthus *et al.* 2015). A potassium ion channel gene, *OsAKT1*, is crucial for tolerance to iron toxicity, as potassium homeostasis affects iron translocation from root to shoot (Wu *et al.* 2019). However, major QTLs or genes underlying tolerance that are effective under iron toxicity in the field have not been identified yet.

Using 18 upland NERICAs (New Rice for Africa) developed as introgression lines with a chromosome segment from CG 14 (*O. glaberrima*) in the genetic background of three Asian rice cultivars, WAB56-104, WAB56-50 and WAB 181-18 (*O. sativa*), and 150 simple sequence repeat (SSR) markers detected several QTLs for nine agricultural traits based on marker polymorphisms including three kinds of alleles of WAB56-104, CG 14 and the other and association analyses (Fukuta *et al.* 2012). Other upland NERICAs and CG 14 were found to be tolerant (Audebert and Fofana 2009, Engel *et al.* 2012, Sahrawat and Sika 2002, WARDA 2002) or moderately tolerant (Asch *et al.* 2005) to iron toxicity. However, the 18 upland NERICAs and their parents have not been assessed for tolerance to iron toxicity, and the genetic mechanisms have not been clarified yet.

The objective of this study was to determine suitable iron concentrations and treatment durations for evaluating the genetic variation in tolerance to iron toxicity in agar nutrient solution, and to characterize tolerance in the 18 upland NERICA varieties and various Indica and Japonica Groups’ varieties. The relationships of the genetic mechanisms between LBS and dry weight are considered as indicators of tolerance to iron toxicity.

## Materials and Methods

### Plant materials

We grew 91 varieties to investigate genetic variation for tolerance to iron toxicity in rice (Table 1): lowland Japonica Group varieties, upland Japonica Group varieties and landraces, Indica Group varieties, and 18 New Rice for Africa (NERICAs) varieties and their parents: CG 14 (*O. glaberrima*, tolerant in iron toxicity field, Sahrawat and Sika 2002), WAB 181-18, WAB 56-50 and WAB 56-104 (*O. sativa*) (Jones *et al.* 1997). Tomita *et al.* (2017) classified them into clusters Ia, Ib and II based on SSR marker polymorphisms, by which cluster Ia corresponded to lowland Japonica Group cultivars, Ib to upland Japonica Group varieties, and II to lowland Indica Group varieties and CG 14.

We used 13 varieties to decide the optimal conditions for assessing genetic variation in tolerance to iron toxicity. Nipponbare (Tolerant in hydroponic culture, Wan *et al.* 2003a), Akihikari (Moderate tolerant in hydroponic culture, unpublished data), Koshihikari and CSC-194 are lowland Japonica Group varieties; CSC-194 is a substitution line with chromosome segments from Indica Group variety, Milyang 23, in the genetic background of Japonica Group variety, Akihikari (Tomita *et al.* 2017). Azucena (Tolerant in hydroponic culture, Wu *et al.*1998) and Owarihatamochi are upland Japonica Group varieties. Milyang 23 (Moderate tolerance in the iron toxicity field, Wang *et al.* 2008), Kasalath (Susceptible in hydroponic culture, Shimizu *et al.* 2005a), IR 64 (Susceptible in hydroponic culture, Wu *et al.*1998 and in iron toxicity field, Wang *et al.* 2008) and US-2 (Fukuta *et al.* 2022) are lowland Indica Group varieties, and three upland NERICAs; NERICA 7, NERICA 10 and NERICA 18, were also included.

### Conditions for evaluation of iron toxicity

We used agar nutrient solution (Wang *et al.* 2008) with minor modifications to evaluate the tolerance of seedlings to iron toxicity. Seeds were soaked at room temperature for four days to germinate. Germinated seeds were placed on mesh floating on 30 L of deionized water in a plastic container for seven days. The resultant seedlings were then grown in a half concentration of Yoshida’s solution (Yoshida *et al.* 1976) for seven days, and the pH of the solution was adjusted to ~5.5 every two days. The seedlings were then placed in Yoshida solution with 0.1% (w/v) agar and 5 mM 2-(N-morpholino) ethanesulfonic acid (MES) buffer (pH 5.5) to constrain the pH (Wiengweera *et al.* 1997). The Yoshida solution comprised NH_4_NO_3_ 1.43 mmol L^–1^, K_2_SO_4_ 0.5 mmol L^–1^, CaCl_2_·2H_2_O 1.0 mmol L^–1^, MgSO_4_·7H_2_O 1.0 mmol L^–1^, NaH_2_PO_4_·2H_2_O 0.32 mmol L^–1^, MnCl_2_·4H_2_O 9 μmol L^–1^, (NH_4_)_6_Mo_7_O_24_·4H_2_O 0.5 μmol L^–1^, H_3_BO_3_ 18.5 μmol L^–1^, CuSO_4_·5H_2_O 0.16 μmol L^–1^, ZnSO_4_·7H_2_O 0.15 μmol L^–1^. In control treatments, iron was supplied as 36 μmol L^–1^ Fe-EDTA. In the investigation of iron concentration and duration of treatment, iron toxicity treatments used FeSO_4_ at 50, 100, 200, or 400 ppm for seven, 14, or 21 days. Fe-EDTA in the control treatment was used because it is a stable and chelated form of iron that ensures consistent iron availability without inducing iron toxicity and allows normal plant growth. FeSO_4_ of iron toxicity treatment was a source of soluble iron to induce iron toxicity because it easily releases ferrous iron in water, which can accumulate to toxic levels in soil-reduced conditions. The solution was exchanged every seven days. The experiments were performed in a naturally sunlit greenhouse at the Japan International Research Center for Agricultural Sciences (JIRCAS), in Tsukuba, Japan, from 21 July to 2 August 2013 to test iron concentration (experiment 1); from 25 July to 15 August 2013 to test treatment duration at 200 ppm Fe (experiment 2); and from 22 November to 13 December 2013 to test treatment duration at 400 ppm (experiment 3). All cultivars were tested in the treatment of 200 ppm for 21 days from 16 August to 20 September 2013.

LBS was evaluated on the last day of the treatment on a scale of 0 (normal) to 9 (dead or dying), following the Standard Evaluation System for rice (IRRI 1996). All plants were collected on the last day and dried for more than 48 h at 80 °C. The dry weight (DW) of shoots and roots of each plant under iron toxicity (Iron-DW) and in the control treatment (Control-DW) was measured. The relative dry weight (RDW) of each plant was calculated as RDW (%) = Iron-DW/Control-DW × 100. The average values of seven plants from experiments 1 to 3 and of three plants in the investigation of all 91 varieties were used as representative data for each. In experiments 1 to 3, we compared the minimum, lower, and upper quartile, median, and mean values among the 13 varieties in each treatment to identify which conditions gave wide variation in these traits. We calculated correlation coefficients (*r*) among these conditions, which showed wide variations to compare the correspondence of the data.

### Cluster analysis

Cluster analysis used the data of LBS, Iron-DW, Control-DW and RDW of the 91 varieties. The data of Iron-DW and Control-DW were added to consider the influences of dray matter production among them. It was carried out using Ward’s hierarchical clustering method (Ward 1963) in JMP v. 7.0.2 software (JMP Statistics and Graphic Guide, SAS Institute, Inc., Cary, NC, USA). The clusters were compared between phenotype data and genotype data (classified by Tomita *et al.* 2017) to reveal their relationship.

### Association analysis between phenotype and genotype data

Phenotype data for LBS, Iron-DW, Control-DW and RDW, and genotype data of 150 SSR markers (McCouch *et al.* 2002) that showed polymorphism among the 18 upland NERICAs (Fukuta *et al.* 2012) were compared by association analysis to detect QTLs for these traits. Single-marker association analysis was carried out in Windows QTL Cartographer v. 2.5 (Wang *et al.* 2011). Associations were estimated by ANOVA between the phenotypic values with two genotypes (WAB56-104 and the other including CG 14 allele), at each SSR marker among 18 NERICAs at Bonferroni adjusted threshold of *P* < 0.05. Positive value of additive effect indicated that the WAB56-104 allele of each QTL (+) increased or (–) decreased the value of each trait.

## Results

### Condition for evaluation of genetic variation for tolerance to iron toxicity

**Fig. 1.**
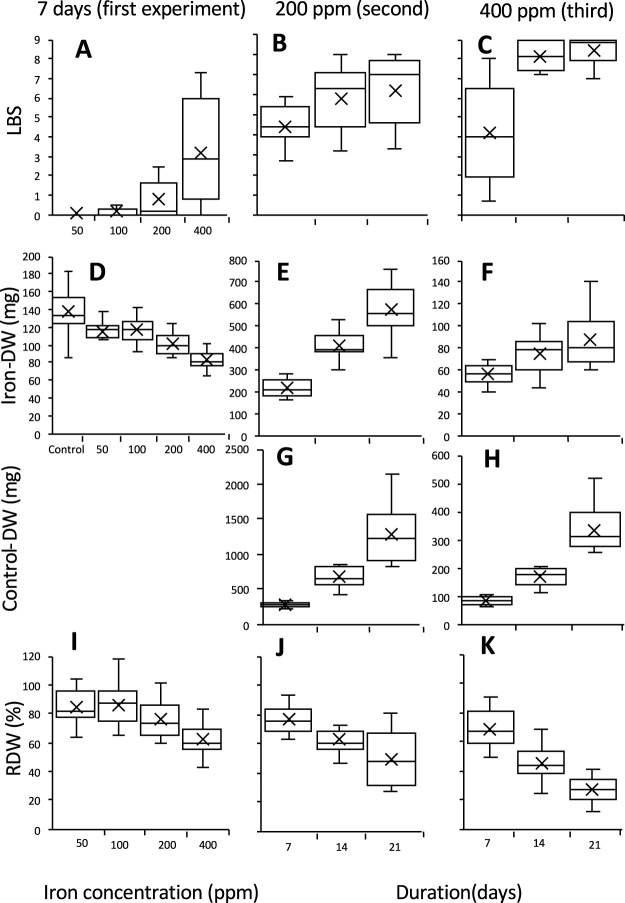
Genetic variations in four traits under iron toxicity treatment among 13 lowland and upland Indica and Japonica Group varieties. (A–C) Leaf bronzing score (LBS), (D–F) dry weight under iron toxicity treatment (Iron-DW), (G, H) dry weight under normal condition (Control-DW), (I–K) relative dry weight (RDW = [Iron-DW/Control-DW] × 100: I–K). Seedlings of each accession were treated at 50, 100, 200, or 400 ppm Fe for 7, 14 or 21 days. Box plots show minimum, lower quartile, median, upper quartile and maximum values of each trait among accessions. × Mean.

At seven days, the medians and means of LBS of the 13 tested varieties increased with iron concentrations (Fig. 1A). Genetic variation was wide at 200 and 400 ppm Fe but narrow at 50 and 100 ppm. The medians and means of Iron-DW and RDW decreased with increasing Fe concentrations, but Iron-DW values at 50 and 100 ppm were similar to those of the control (Fig. 1D, 1I). These results indicate that 50 and 100 ppm were not sufficient for the evaluation of genetic variation in LBS and RDW.

The 13 varieties were treated again for seven, 14 or 21 days at 200 or 400 ppm Fe. At 200 ppm, the medians and means of LBS, Iron-DW, and Control-DW increased and those of RDW decreased as duration increased (Fig. 1B, 1E, 1G, 1J). The variation in LBS was wider at 14 and 21 days than at seven days. All variations widespread at 21 days. At 400 ppm also, the medians and means of LBS, Iron-DW and Control-DW increased and RDW decreased as duration increased (Fig. 1C, 1F, 1H, 1K). Variations of LBS and RDW were the widest at seven days, but those of Iron-DW and Control-DW were the widest at 21 days. Therefore, 21 days at 200 ppm and seven days at 400 ppm were determined as most suitable for evaluation of variation in tolerance to iron toxicity.

To compare the results of these two conditions, we investigated the correlation coefficients of each trait (Supplemental Table 1). Values ranged from –0.43 to +0.79, and only LBS had a significant positive correlation (*r* = 0.79). Therefore, the variations in Iron-DW, Control-DW, and RDW did not correspond between the two conditions. Moreover, the correlations between Iron-DW and Control-DW decreased at both concentrations as durations increased (data not shown). These results suggest that the contradictory results among the three traits are due to differences in early growth before treatment.

The results were also compared between the iron toxicity field tolerant cultivar Milyang 23 and the susceptible variety IR 64 (data not shown). There were higher LBS, 4.1, for Milyang 23 at 21 days of 200 ppm, 2.8 at seven days of 400 ppm, and 7.0 for IR 64 at 21 days of 200 ppm, and 7.0 at seven days of 400 ppm. For RDW, Milyang 23 and IR 64 were 74% and 50% at seven days of 400 ppm. On the other hand, at 21 days of 200 ppm, Milyang 23 and IR 64 were 28% and 47%, respectively, indicating the reversal results.

### Genetic variations in tolerance to iron toxicity

**Fig. 2.**
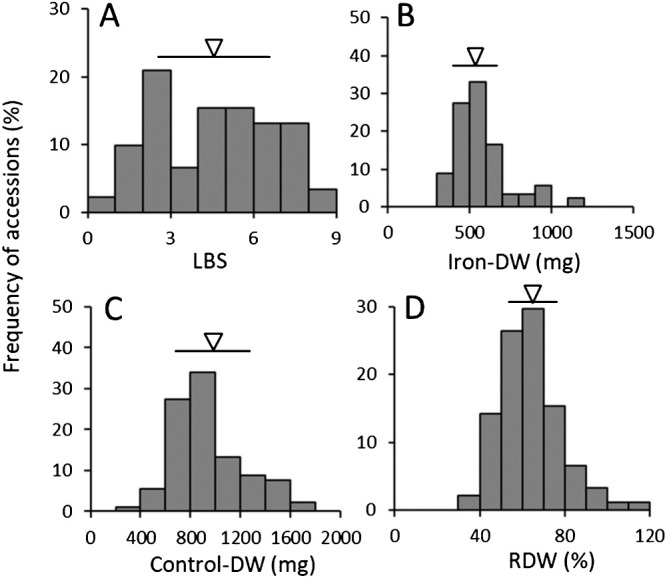
Genetic variations of four traits under iron toxicity treatment among 91 lowland and upland Indica and Japonica Group varieties and 18 upland NERICAs. (A) LBS, (B) Iron-DW, (C) Control-DW, (D) RDW treated at 200 ppm Fe for 21 days. 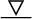 mean and SD.

**Fig. 3.**
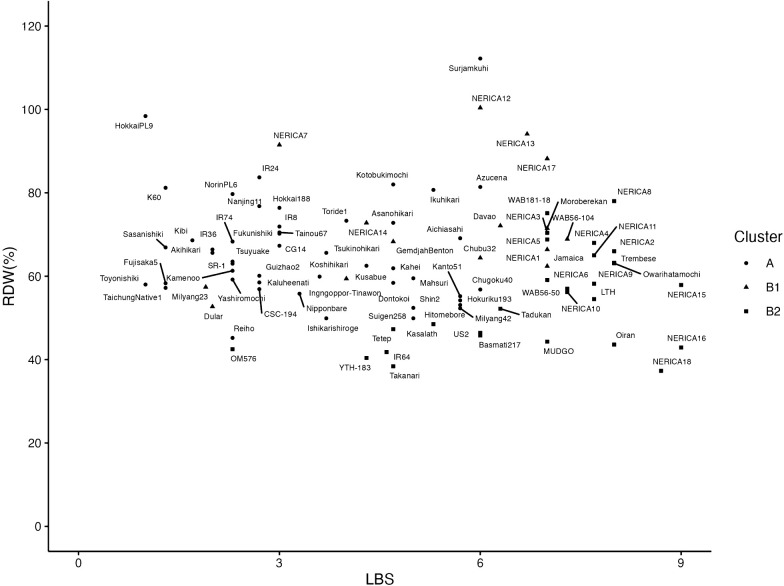
Relationship between LBS and RDW among 91 varieties under the treatment of Fe concentration, 200 ppm, and duration of 21 days. Correlation coefficient between LBS and RDW: *r* = –0.10 (not significant at *P* = 0.05). These accessions were classified into three groups; A (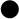), B1 (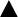), and B2 (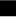) by cluster analysis using data of LBS, Iron-DW, Control-DW, and RDW.

To clarify genetic variations in LBS and DW without the effects of differences in the early growth of seedlings before treatment, we grew all 91 cultivars for 21 days at 200 ppm Fe. Values varied widely: LBS from 1.0 to 9.0, Iron-DW from 312 to 1137 mg, Control-DW from 385 to 1766 mg, and RDW from 37% to 112% (Figs. 2, 3, Table 2). The distributions of LBS had two peaks, at 2–3 and 4–6 (Fig. 2A). The other three traits had continuous one-peak distributions (Fig. 2B–2D).

There were no significant correlations between LBS and the other three traits; Control-DW, Iron-DW and RDW (Table 3). Iron-DW had significant positive correlations with both Control-DW and RDW, and Control-DW had a significant negative correlation with RDW. There were moderate correlations between Iron-DW and RDW, as well as between Control-DW and RDW.

### Classification of rice accessions

Based on the four traits; LBS, Control-DW, Iron-DW and RD the 91 varieties were classified into clusters A, B1 and B2 (Fig. 3, Tables 1, 2). Cluster A had 46 varieties; the mean LBS was 3.4, the lowest among the three clusters. Iron-DW and Control-DW were also the lowest, and RDW was intermediate between B1 and B2. B1 had 13 varieties; the mean LBS was intermediate between A and B2, and the other traits had the highest values among the three clusters. B2 held the remaining 32 varieties; the mean LBS was the highest, RDW was the lowest, and Iron-DW and Control-DW were intermediate between those of A and B2. Therefore, the varieties of cluster A were tolerant, with the lowest LBS, but both Iron-DW and Control-DW were low. LBS of B1 was intermediate, and B1 was the most tolerant in DW, with the highest values of Iron-DW, Control-DW and RDW among the three clusters. B2 had also high DW but was susceptible to iron toxicity in LBS and DW.

Cluster A held mainly lowland Indica or Japonica Groups’ cultivars (Tomita *et al.* 2017). B1 and B2 held both lowland and upland Indica and Japonica Groups’ cultivars. B1 held five upland NERICAs; NERICA 7, 12, 13, 14 and 17. B2 held the other 13 NERICAs and the three *O. sativa* parents. Thus, upland NERICAs and three parents were categorized into different groups of tolerance to iron toxicity.

### QTLs using upland NERICAs

LBS, Iron-DW, Control-DW and RDW varied widely in the 18 upland NERICAs and their four parents (Fig. 2). The three recurrent parents, WAB56-104, WAB56-50 and WAB181-18, are high LBS (>6). NERICA 7, NERICA 14 and CG 14 had low values of LBS (<5), and NERICA 15, NERICA 16 and NERICA 18 had high values (>8). RDWs of NERICA 7, NERICA 12, NERICA 13 and NERICA 17 were high but those of NERICA 6, NERICA 9, NERICA 10, NERICA 16 and NERICA 18 were low. Iron-DWs and Control-DWs in NERICA 7 and NERICA 17 were high. Iron-DWs in NERICA 12 and NERICA 13 were almost as high, but those of Control-DW were moderate. Iron-DWs in NERICA 6, NERICA 8, NERICA 9, NERICA 10, NERICA 15, NERICA 16 and NERICA 18 were low. Control-DWs in NERICA 8 were low, and those in NERICA 16 and NERICA 18 were high. These results place the high-RDW lines into two types: NERICA 7 and NERICA 17 with high Iron-DW and Control-DW, and NERICA 12 and NERICA 13 with high Iron-DW and intermediate Control-DW; and place the low-RDW lines into two types: NERICA 6, NERICA 8, NERICA 9 and NERICA 10 with low Iron-DW and low or intermediate Control-DW, and NERICA 16 and NERICA 18 with high Control-DWs and low Iron-DWs.

**Fig. 4.**
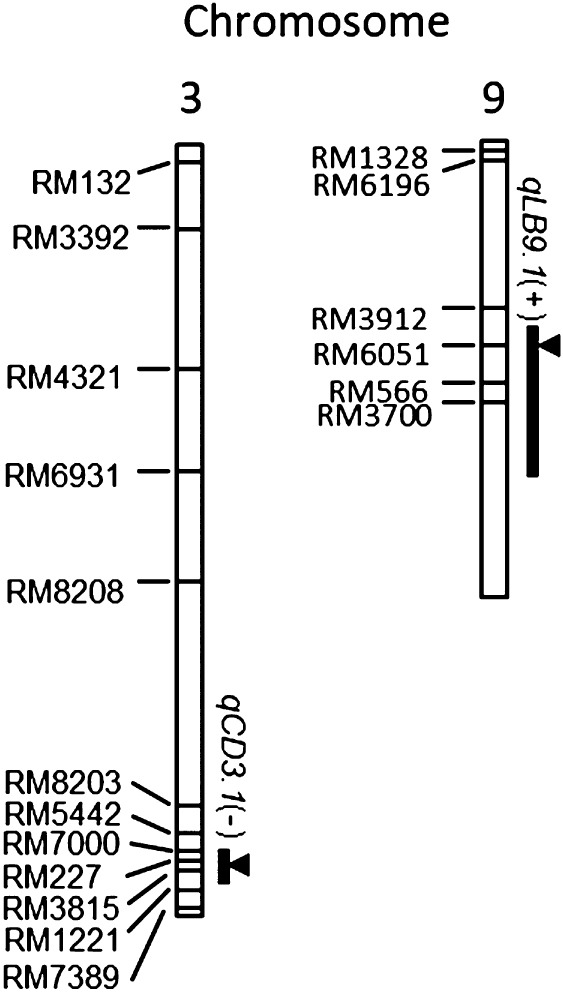
QTLs for LBS (*qLB*), and Control-DW (*qCD*) detected under iron toxicity treatment in association analyses using 18 upland NERICA cultivars. Association analyses were performed between the genotypes of 150 SSR markers (Fukuta *et al.* 2012). The variations in the four traits among 18 upland NERICAs at a Bonferroni adjusted threshold of *P* ≤ 0.05. Horizontal bars on each chromosome indicate marker positions. 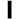 QTLs detected; 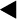 highest *F*-value at each QTL. “+”, WAB 56-104 allele increases trait values; “–”, CG 14 allele.

We detected two QTLs for the two traits among the 18 NERICAs—LBS and Control-DW—on chrs. 9 and 3, respectively (Fig. 4, Table 4). For the QTL of LBS, *qLB9*, was, the *O. sativa* allele increased the value. *F*-value of QTL for LBS was 38.7, and *R*^2^ value was 70.7%. The QTL for Control-DW, *qCD3.1*, was detected on chr. 3; the CG 14 allele of the QTL increased the value. Therefore, low LBS in several NERICAs due to major genetic factor(s) on chr. 9 from CG 14. The genetic factor on chr. 3 from CG 14 might contribute to higher DW of several NERICAs under the control condition.

## Discussion

### Condition of iron toxicity

Our modified use of 0.1% (w/v) agar nutrient solution (Wang *et al.* 2008) with 5 mM MES buffer suppressed sudden drops in pH (data not shown). This method allowed us to determine that 21 day-duration in 200 ppm FeSO_4_ was appropriate to reveal the variation in tolerance of rice varieties to iron toxicity (Fig. 1). It also avoided the effects of differences in the early growth of seedlings. Previous studies for screening tolerance using hydroponic solutions (Asch *et al.* 2005, Dufey *et al.* 2009, 2015a, 2015b, Elec *et al.* 2013, Engel *et al.* 2012, Fukuda *et al.* 2012, Matthus *et al.* 2015, Ouyang *et al.* 2007, Shimizu *et al.* 2005a, 2005b, Wan *et al.* 2003a, 2003b, Wang *et al.* 2008, Wu *et al.* 1998, 2014, Yamauchi *et al.* 1982) used wide ranges of iron concentrations (100–3000 ppm) and treatment durations (2–28 days). Several showed that tolerance in some cultivars changed with conditions (Fukuta and Wissuwa 2011, Mahender *et al.* 2019). For example, Suakoko 8 showed leaf bronzing immediately after iron toxicity treatment (Yamauchi *et al.* 1982) but it survived for a long time (Audebert and Fofana 2009, Elec *et al.* 2013); Elec *et al.* (2013) suggested that it could accumulate iron in leaf blade tissues. Thus, differences in response to stress might give contradictory results. We also found contradictory results in Iron-DW between 200 ppm for 21 days and 400 ppm for seven days (Supplemental Table 1), in which the correlations between Iron-DW and Control-DW decreased as the duration increased (data not shown). Elec *et al.* (2013) reported that resistance to iron toxicity is better correlated with field grain yield in long-term treatment than in short-term treatment. Therefore, to match the results of agar nutrient culture with those of field culture, long-term treatment is better. The use of 200 ppm for 21 days revealed wide variation in tolerance traits without effects of early growth before starting the treatment and might correspond well with growth in field conditions. As Iron-DW and Control-DW were highly correlated in the investigation of 91 varieties (Table 3), varietal differences should be analyzed in detail. Moreover, the results were also compared between the iron toxicity field tolerant variety, Milyang 23 and the susceptible variety, IR 64 (data not shown). Milyang 23 showed lower LBS than those of IR 64 under both 21 days of 200 ppm and seven days of 400 ppm. On the other hand, the results of RDW showed a reversal with Milyang 23 at 28% and IR 64 at 47% at 21 days of 200 ppm; 74% and 50% at 7 days of 400 ppm. At 21 days of 200 ppm, Control-DW for Milyang 23 was remarkably higher than those of 14 days, indicating that the increase of Control-DW reduced RDW for Milyang 23. This reversal did not occur when 91 varieties were evaluated (Fig. 3). These results suggest that variation of Control-DW may affect the reproducibility of the results. In this study, experiment 2, which showed remarkably higher Control-DW at 21 days of 200 ppm, was conducted from July 25 to August 15, 2013, and evaluation of 91 varieties was from August 16 to September 20 in a naturally sunlit greenhouse in Japan. The environmental conditions of Experiment 2 included higher temperature and more solar radiation. Under the better condition among varieties in the young growth stage, the differences of Control-DWs between Indica Group high-yielding varieties Milyang 23 and IR 64 might be demonstrated well.

Therefore, the value of Control-DW should be considered when using RDW to select tolerant varieties. Many studies have used LBS to indicate tolerance to iron toxicity. Significant correlations between LBS and grain yield in the field (Audebert and Fofana 2009, Elec *et al.* 2013, Sikirou *et al.* 2015, Yamauchi 1989) or DW under hydroponic conditions (Dufey *et al.* 2009, 2012, 2015a, 2015b, Wan *et al.* 2003b, Wu *et al.* 1998) have been reported. However, in our study the wide genetic variations in LBS and DW among all 91 varieties were not correlated (Figs. 2, 3, Table 3), therefore, these traits might be controlled by different genetic factors. The main mechanisms responsible for tolerance to iron toxicity are oxidation at the roots and Fe selectivity, retention of Fe in roots and stems, and symplastic tissue tolerance (Becker and Asch 2005). Leaf bronzing is a result of the accumulation of oxidized polyphenols in the leaf blades after loss of symplastic tissue tolerance. Therefore, variations in leaf bronzing and DW under iron toxicity might not result from the same mechanisms. To clarify the genetic mechanisms responsible for tolerance to iron toxicity or to develop tolerant materials, it will be useful to isolate the factors responsible for both LBS and DW. In addition, to further clarify the relation between phenotype and tolerance mechanisms, the relation of phenotype with Fe uptake and homeostasis, oxidative power of roots, or reaction for oxidative stress should be analyzed.

Wide variations of iron tolerance and no correlation between LBS and RDW were found among 91 varieties using the agar nutrient solution (Fig. 3, Table 3). Among them, Hokkai PL9 showed a low value of LBS and a high RDW and was categorized as a tolerant variety. In contrast, Oiran, NERICA 16 and NERICA 18 had high LBS and low RDW and these varieties were sensitive in both traits. Surjamkuhi and NERICA 12 showed high LBS and RDW. Reiho and OM576 showed low LBS and RDW. These results indicated that the genetic mechanisms between LBS and RDW were different and independent. It means that the breeding for iron toxicity will be able to carry out for targeting two traits, LBS and RDW, independently, and it will be possible to accumulate genes from both traits. Among the 91 varieties, each of the nine groups defined in the matrix of three genotype clusters × three phenotypic groups included at least one variety (Table 1). Thus, each genotype cluster has wide genetic variations in tolerance to iron toxicity. Many lowland Japonica Group varieties were classified into the low-LBS group (A), with few high-RDW varieties. Japonica Group varieties for lowland tended to be classified into high DW groups (B1 and B2), but they showed wide variation in RDW. Most of the Indica Group varieties were divided into A and B2, including bronzing-tolerant accessions, and bronzing-sensitive and low RDW ones. These results confirm that the variations in tolerance were controlled by different genetic mechanisms between LBS and DW, and that the differentiation between Japonica and Indica Groups, adaptation to lowland or upland, and degree of improvement affect each mechanism. Engel *et al.* (2012) evaluated 21 varieties in hydroponic culture and reported that Indica Group varieties and *O. glaberrima* had wide variation in LBS under iron toxicity; this supports our results, but they also stated that Japonica Group varieties tended to have high LBS. We assessed a wide range of variation in tolerance in Japonica Group varieties, using a large number of varieties (both lowland and upland, improved and landrace accessions) than those of Engel *et al.* (2012). In the degraded lowland fields of Japan over 60 years ago, *akagare* and *akiochi* diseases caused serious problems with the appearance of tiny brown spots on the leaves and reduced yields owing to oxidative stress due to excess ferrous iron and low potassium, magnesium, manganese, and silica (Baba and Tajima 1960, Park and Tanaka 1968). Therefore, the wide variation in LBS in Japonica Group varieties might explain the differentiation of symptoms in those degraded lowland fields. On the other hand, the wide variation in DW in Indica Group varieties did not show remarkable differentiation. Therefore, these variations might be due to the accumulation of minor genetic factors.

Among 18 upland NERICAs, many NERICAs showed high LBS, except for NERICA 7 and NERICA 14 (Fig. 3). Also, their three recurrent parents indicated high LBS. These parents are rainfed upland varieties (Fukuta *et al.* 2012). Consequently, they might be susceptible to stress in reducing soils, including iron toxicity. The high LBS of many NERICAs may succeed the susceptible genetic backgrounds of these parents. Among the 18 upland NERICAs, two significant QTLs for LBS on chr. 9 and for Control-DW on chr. 3 were detected (Fig. 4, Table 4). Many QTLs have been reported for LBS in previous studies (Asch *et al.* 2005, Dufey *et al.* 2009, 2012, Elec *et al.* 2013, Engel *et al.* 2012, Fukuda *et al.* 2012, Matthus *et al.* 2015, Shimizu *et al.* 2005a, Wan *et al.* 2003a, 2003b, Wang *et al.* 2008, Wu *et al.* 1998, 2014, Yamauchi *et al.* 1982), and some alleles of glaberrima have been identified that reduce LBS (Diop *et al.* 2020, Dufey *et al.* 2015b, Melandri *et al.* 2021). However, *qLB9.1*, the *O. glaberrima* allele of which decreased LBS, was a novel QTL. However, our association analyses might be compromised by missing data and overcounting (Fukuta *et al.* 2012). Therefore, the QTLs identified here need to be confirmed in hybrid populations by co-segregation analysis. Also, *qCD3.1*, where a QTL on chr. 3 for Control-DW was found, has been reported; in a previous study the allele of *O. glaberrima* was associated with grain yield under iron toxicity (Melandri *et al.* 2021). Several QTLs related to iron toxicity tolerance have also been reported in this region of chr.3 with *O. sativa* allele for LBS and plant height (Wan *et al.* 2003a), iron content of shoot (Shimizu *et al.* 2005a), DW (Dufey *et al.* 2009, Wan *et al.* 2003b). Therefore, although this region was not detected in Iron-DW in this study only in Control-DW, it is a significant QTL region that may also play a role in biomass and yield under iron toxicity. For Iron-DW and RDW, no significant QTL could be detected in this study. This suggests that the variation in DW under iron toxicity might be due to multiple QTLs with small effects.

We clarified wide genetic variation in rice germplasm for LBS and DW under iron toxicity treatment in agar nutrient solution with 200 ppm iron over 21 days. The method, the information on cultivars, and the QTLs with favorable allele of *O. glaberrima* detected here will be valuable in breeding of cultivars tolerant to iron toxicity in the field. Our findings suggest that tolerance to iron toxicity is due to many genetic factors with small effects. Although we expected that these genetic analyses would be difficult, it may be possible to accumulate these genetic factors by conventional breeding, according to the results of classification and QTL detection by association analysis in upland NERICA cultivars.

## Author Contribution Statement

AT carried out this research work and wrote the first manuscript. YF conducted and selected the rice materials of this study. J-P Tanaka and MW supported and made suggestions for the evaluation method of iron toxicity.

## Supplementary Material

Supplemental Table

## Figures and Tables

**Table 1.** Relationship between the cluster groups for phenotype data under iron toxicity treatment and for genotype data of SSR markers

Cluster groups based on genotype data		Name and No. of accessions (%)			Total
		Cluster groups based on phenotype data under iron toxicity treatment			
		A	B1	B2	
I	a	Aichiasahi, Akihikari, Asanohikari, Chugoku 40, CSC-194, Dontokoi, Fujisaka 5, Fukunishiki, Hitomebore, Hokkai 188, Hokkai PL9, Ikuhikari, K60, Kibi, Kotobukimochi, Kusabue, Nipponbare, Norin PL6, Reiho, Sasanishiki, Shin 2, Tainou 67, Toride 1, Toyonishiki, Tsukinohikari, Tsuyuake,Yashiromochi	Koshihikari	OM576, Ishikarishiroge	
	Sum	27 (90.0)	1 (3.3)	2 (6.7)	30 (100.0)
	b	Azucena, Kamenoo	Chubu32, Davao, Gemdjah Benton, Ingngoppor-Tinawon, Kahei, NERICA7, NERICA12, NERICA13, NERICA14, NERICA17	Jamaica, LTH, Moroberekan, NERICA1, NERICA2, NERICA3, NERICA4, NERICA5, NERICA6, NERICA8, NERICA9, NERICA10, NERICA11, NERICA15, NERICA16, NERICA18, Oiran, Owarihatamochi, Trembese, WAB181-18, WAB56-50, WAB56-104	
	Sum	2 (5.9)	10 (29.4)	22 (64.7)	34 (100.0)
II		CG 14, Guizhao 2, Hokuriku 193, IR 8, IR 24, IR 36, IR 74, Kaluheenati, Kanto51, Mahsuri, Milyang 42, Nanjing 11, SR-1, Surjamkuhi, Suweon 258, Taichung Native 1, Tetep	Dular, Milyang 23	Basmati 217, IR 64, Kasalath, MUDGO, Tadukan, Takanari, US-2, Kachibai (YTH183)	
	Sum	17 (63.0)	2 (7.4)	8 (29.6)	27 (100.0)
Total		46 (50.5)	13 (14.3)	32 (35.2)	91 (100.0)

91 varieties were classified by cluster analysis into clusters A, B1 and B2 based on phenotype data LBS, Iron-DW, Control-DW and RDW using Ward’s hierarchical clustering method. The classification of clusters Ia, Ib and II, based on DNA polymorphism data (Tomita *et al.* 2017), was compared with phenotype clusters. Ia included mainly improved lowland Japonica Group varieties, Ib included mainly upland Japonica Group varieties, and II included mainly Indica Group varieties.

**Table 2.** Genetic variation in four traits among three clusters

Cluster group	No of accessions	Traits			
		LBS	Iron-DW (mg)	Control-DW (mg)	RDW(%)
A	46	3.4 ± 1.6^a^	495.2 ± 86.4^a^	764.6 ± 154.3^a^	66.3 ± 12.9^b^
		(1.0–6.0)	(312.4–688.0)	(384.5–1209.5)	(45.2–112.2)
B1	13	4.6 ± 1.6^b^	895.3 ± 133.4^c^	1258.3 ± 258.8^c^	73.2 ± 14.7^b^
		(1.9–7.0)	(673.3–1136.5)	(917.7–1766.4)	(52.7–100.4)
B2	32	6.8 ± 1.5^c^	559.3 ± 86.5^b^	1039.9 ± 291.0^b^	56.6 ± 11.5^a^
		(2.3–9.0)	(395.1–745.9)	(506.5–1681.9)	(37.3–78.0)
Total	91	4.8 ± 2.2	574.9 ± 164.1	931.9 ± 291.3	63.7 ± 14.0
		(1.0–9.0)	(312.4–1136.5)	(384.5–1766.4)	(37.3–112.2)

A total of 91 varieties were classified into clusters A, B1 and B2 based on phenotype data of LBS, Iron-DW, Control-DW and RDW. Values are means ± SD. Within a column, values with the same letter are not significantly different at *P* = 0.05 by Tukey’s test.

**Table 3.** Correlation coefficients among four traits of 91 varieties

Traits	LBS	Iron-DW	Control-DW
Iron-DW	0.04	–	–
Control-DW	0.11	0.68***	–
RDW	–0.10	0.26*	–0.49***

Significant at * *P* = 0.05 and ** 0.001.

**Table 4.** QTLs detected by association analysis using 18 NERICAs

Trait	QTL	Chr.	Position (Mbp)	Marker name	*F*	*P* value	*R*^2^ (%)	Additive effect	Positive allele
LBS	*qLB9.1*	9	12.8	*RM6051*	38.7	0.00001	70.7	1.96	WAB56-104
Control-DW	*qCD3.1*	3	34.8	*RM3815*	21.0	0.00030	56.8	–167.19	CG 14

A total of 18 upland NERICAs were used to detect QTLs for the four traits. Using genotype data (Fukuta *et al.* 2012) and the values of four traits in each variety, associations between them were detected at *P* = 0.01. Marker name indicates the QTL-linked marker with the highest *F* score. Positive value of additive effect indicates for increasing the trait’s value with WAB56-104 allele, while negative value was with the allele of CG 14.
