# Supplementary material for: Genetic variation in leaf bronzing and dry matter production of rice varieties as indicators of tolerance to iron toxicity
Source: Breed Sci. 2025 Jul 31;75(4):292–302. doi: 10.1270/jsbbs.24081 (PMC13051631; doi:10.1270/jsbbs.24081)
Supplement: Supplementary file 1 — Supplemental Table [file 75_292_s1.pdf]

doi: 10.1270/jsbbs.24081

Supplemental Table 1. Relationships between two different treatments of iron toxicity in 14 varieties

Conditions and traits		*r* value (200 ppm, 21 days)				
		LBS		Iron-DW	Control-DW	RDW
(400 ppm, 7 days)	LBS	0.79	**	−0.27	0.13	−0.35
	Iron-DW	0.08		0.01	0.46	−0.31
	Control-DW	0.35		0.02	0.49	−0.43
	RDW	−0.23		0.05	−0.13	0.22

Corelation coefficients (*r*) between the two conditions were calculated among accessions.
